# Modulation of blood redox status by the progression of induced apical periodontitis in rats

**DOI:** 10.3389/fphys.2023.1214990

**Published:** 2023-09-05

**Authors:** Deborah Ribeiro Frazão, Paulo Fernando Santos Mendes, Daiane Claydes Baia-da-Silva, João Daniel Mendonça de Moura, Vinicius Ruan Neves dos Santos, José Mario Matos-Sousa, Gabriela de Souza Balbinot, Douglas Magno Guimarães, Fabrício Mezzomo Collares, Rafael Rodrigues Lima

**Affiliations:** ^1^ Laboratory of Functional and Structural Biology, Institute of Biological Sciences, Federal University of Pará (UFPA), Belém, Brazil; ^2^ Dental Material Laboratory, School of Dentistry, Federal University of Rio Grande do Sul (UFRGS), Porto Alegre, Rio Grande do Sul, Brazil; ^3^ Centro Universitário do Para (CESUPA), Belém, Pará, Brazil

**Keywords:** periapical periodontitis, oxidative stress, wistar rats, microCT, inflammation

## Abstract

This study aimed to investigate if apical periodontitis in different periods changes systemic levels of the antioxidant and pro-oxidant parameters in Wistar rats. Twenty-four rats were randomly allocated into healthy animals, apical periodontitis at 14 days (AP14) and apical periodontitis at 28 days (AP28). The first mandibular molars were accessed in the AP groups, and the pulp chamber was exposed to the oral environment, inducing the apical lesion. After 14 and 28 days, the animals were anesthetized, euthanized, and hemimandibles were collected for micro-computed tomography (micro-CT) analysis to measure lesion volume, bone volume (BV), percent of bone to total tissue volume (BV/TV), trabecular thickness (Tb.Th), trabecular number (Tb.N), and trabecular space (Tb.Sp). A histological examination of the remaining bone was also performed. Finally, blood samples were collected for oxidative biochemistry analysis, investigating glutathione (GSH), Trolox equivalent antioxidant capacity (TEAC), and lipid peroxidation (TBARS). The lesion volume was greater at 28 than at 14 days, as shown by micro-CT. AP14 and AP28 had decreased BV and Tb.Th, but only AP28 showed a reduction in BV/TV. Tb.N and Tb. Sp were increased in apical periodontitis at 28 days. In the histopathological analysis, AP14 had focal regions of moderate mononuclear inflammatory infiltrate, and AP28 had an intense inflammatory infiltrate with bacterial colonies. In the biochemical evaluation, GSH, TEAC, and TBARS were increased after 14 days. However, GSH returned to control levels, TEAC was similar to AP14, and TBARS increased significantly after 28 days. Therefore, the oxidative biochemistry response was modulated according to the progression of periapical damage. After 14 days, the organism could still react to the injury. However, at 28 days, the antioxidant response decreased, associated with an increase in TBARS.

## 1 Introduction

Apical periodontitis (AP) is an inflammatory disease that arises due to an infectious process within the root canal system of a tooth. This condition results in persistent inflammation and bone resorption in the apical region (Sehirli et al., 2019). The initial inflammatory changes in the pulp are primarily a consequence of microbial infection (Segura-Egea et al., 2015; Tibúrcio-Machado et al., 2021) As the infection progresses, there is an influx of polymorphonuclear leukocytes (PMNs) and monocytes into the pulp, leading to an intensified cellular infiltrate (Stashenko et al., 1998; Cosme-Silva et al., 2020). The interconnection between the root canal system and periradicular tissues through the apical foramen and foramina enables the spread of intracanal infections to these tissues (Sehirli et al., 2019). Consequently, pulp infection leads to chronic inflammation and bone resorption in the apical region, resulting in a periapical lesion (Sehirli et al., 2019; Tibúrcio-Machado et al., 2021). Persistent aggressive stimuli can induce tissue damage in the host (Aksoy et al., 2019; Lopes and Siqueira, 2020), due to the release of inflammatory cytokines that stimulate bone-resorbing cells, such as osteoclasts (Siqueira and Rôças, 2007; Cosme-Silva et al., 2020).

In addition to its local effects, apical periodontitis may contribute to the systemic immune response through a variety of pathways, including changes in interleukin expression (Baeza et al., 2016; Garrido et al., 2019; Haug and Marthinussen, 2019; Sirin et al., 2021), immunoglobulins (Garrido et al., 2019; Sirin et al., 2021), C-reactive protein (Garrido et al., 2019; Sirin et al., 2019), and biochemical factors (Chen et al., 2021; Bakhsh et al., 2022). Consequently, there is evidence suggesting that AP contributes to increased systemic inflammation (Inchingolo et al., 2013; Zhang et al., 2016; Hernández-Ríos et al., 2017; Georgiou et al., 2021; Poornima et al., 2021; Bakhsh et al., 2022; Niazi and Bakhsh, 2022) as chronic infections activate inflammatory mediators that exert systemic effects (Gomes et al., 2013; Niazi and Bakhsh, 2022).

The generation of excessive free radicals (FR) and depletion of antioxidants by pro-inflammatory mediators can lead to several cellular adverse effects (Khansari et al., 2009), triggering or exacerbating the pathogenesis of various diseases FRs are characterized by unpaired electrons centered on oxygen or nitrogen atoms, resulting in the generation of reactive oxygen species (ROS) or nitrogen species (RNS) (Birben et al., 2012; Sies, 2015; Sies et al., 2017). While free radicals play crucial biological roles, an imbalance between their generation and the action of antioxidants, which are the primary defense against pro-oxidant damage, can result in oxidative stress (OS) (Palacios et al., 2015; Rodrigues de Araujo et al., 2018). This oxidative stress can cause lipid peroxidation of membranes and damage enzymes, proteins, carbohydrates, RNA, and DNA (Barreiros et al., 2006; Miricescu et al., 2011).

The association between blood redox and inflammation is complex. Chronic inflammation is characterized by persistent immunological activation and the production of inflammatory cytokines. Inflammatory cells such as macrophages, neutrophils, and eosinophils contribute to inflammation by generating inflammatory cytokines and chemokines (Khansari et al., 2009). Importantly, as part of the immunological response, these immune cells can produce ROS. The increased generation of reactive oxygen species (ROS) at the site of inflammation results in endothelial dysfunction and tissue injury. The vascular endothelium plays a crucial role in the transmigration of macromolecules and inflammatory cells from the bloodstream to the surrounding tissue (Mittal et al., 2014). When the equilibrium between ROS production and antioxidant defense systems is disrupted, excessive ROS can further exacerbate inflammation by activating pro-inflammatory signaling pathways and damaging tissues (Khansari et al., 2009; Mittal et al., 2014).

Animal models have been extensively used in dental research to investigate the apical and periapical repair processes, partly due to their anatomical and physiological similarities with humans. The utilization of these models facilitates the establishment of predictable periradicular lesions after experimental pulp exposure (Stashenko et al., 1992), with rapid outcomes due to their high growth rate (Wang et al., 2010). While ferrets (Verma et al., 2017), dogs (Torabinejad et al., 1995), and monkeys (FABRICIUS et al., 1982) have been reported as suitable animal models (Richert et al., 2022), rats remain the most used (Kakehashi et al., 1965; Kalatzis-Sousa et al., 2017). Although animal models have their limitations in studying systemic parameters and oral infection, they do provide a means of investigating the underlying mechanisms of such diseases and utilizing methodologies that would otherwise be unfeasible in human studies (Cintra et al., 2021). Thus, rats were selected for the present investigation.

Rat experiments have a rich history in the study of apical periodontitis and have played a crucial role in elucidating the complex cellular and molecular events involved in its development and progression. For instance, a study examined the kinetics of macrophages and lymphoid cells during the induction of experimental periapical lesions in rat molars (Kawashima et al., 1996). This quantitative immunohistochemical investigation shed light on the dynamic cellular processes that contribute to the formation and progression of AP. Moreover, animal experiments allow researchers to investigate the interactions between apical lesions and systemic conditions, such as cardiovascular diseases (Brilhante Wolle et al., 2012) and diabetes (Prieto et al., 2017). In addition, rat models have facilitated the exploration of potential therapeutic approaches, including the use of adjuvant substances that can mitigate bone resorption and promote tissue repair (Aksoy et al., 2019; Sehirli et al., 2019).

This study hypothesized that the progression of apical periodontitis is associated with increasing oxidative damage and subsequently decreasing the antioxidant response. Therefore, this study aimed to investigate if apical periodontitis at different phases modulates the oxidative biochemistry response in Wistar rats.

## 2 Material and methods

### 2.1 Animals

Male Wistar rats (90 days old; N = 24), obtained from an animal facility (Federal University of Pará), were allocated randomly to cages with controlled NUVITAL^®^ food (3 pellets/animal) and water *ad libitum*. The composition of the pellets are ground whole corn, soy bran, wheat bran, calcium carbonate, bicalcium phosphate, sodium chloride, vitamin-mineral premix, and amino acids. A 12 h light/dark cycle (lights on 7 AM) and temperature control (25°C ± 1°C) were used. All procedures were approved by the Ethics Committee on Experimental Animals of the Federal University of Pará (under number 7599261120), following the Guide for the Care and Use of Laboratory Animals recommendations (National Research Council US, 2011) and the ARRIVE guidelines (du Sert et al., 2020).

### 2.2 Experimental procedures

The animals were divided randomly into a control group (sham), that is, healthy animals without lesion induction (n = 8), apical periodontitis at 14 days (AP14, n = 8), and apical periodontitis at 28 days group (AP28, n = 8). Animal numbers per group were obtained based on Prieto et al. (2017) study through G*Power software (Statistical Power Analyses 3.1.9.2); the effect size was 1.923, α err prob 0.05, and a power of 0.95. On the first day of the experiment, apical periodontitis groups were submitted to experimental apical periodontitis induction.

The methods for inducing apical periodontitis were based on the studies of Sehirli et al. (2019); Aksoy et al. (2019) with some adaptations. The animals were anesthetized by intraperitoneal anesthesia [xylazine 2% (8 mg/kg) plus ketamine 10% (90 mg/kg)] and placed on a surgical table. After that, the pulp of the left and right mandibular first molars was exposed by a carbide #1/4 burr (KaVo Dental, Biberach an der Riß, German). Then, a C-Pilot #10 file was inserted into the tooth to confirm access to the root canals and stimulate pulpal bleeding. The exposed teeth were left open to the oral environment to develop apical lesions for 14 days and 28 days. To minimize pain and discomfort, a daily dose of 100 mg/kg dipyrone was administered subcutaneously for 3 days.

**FIGURE 1:**
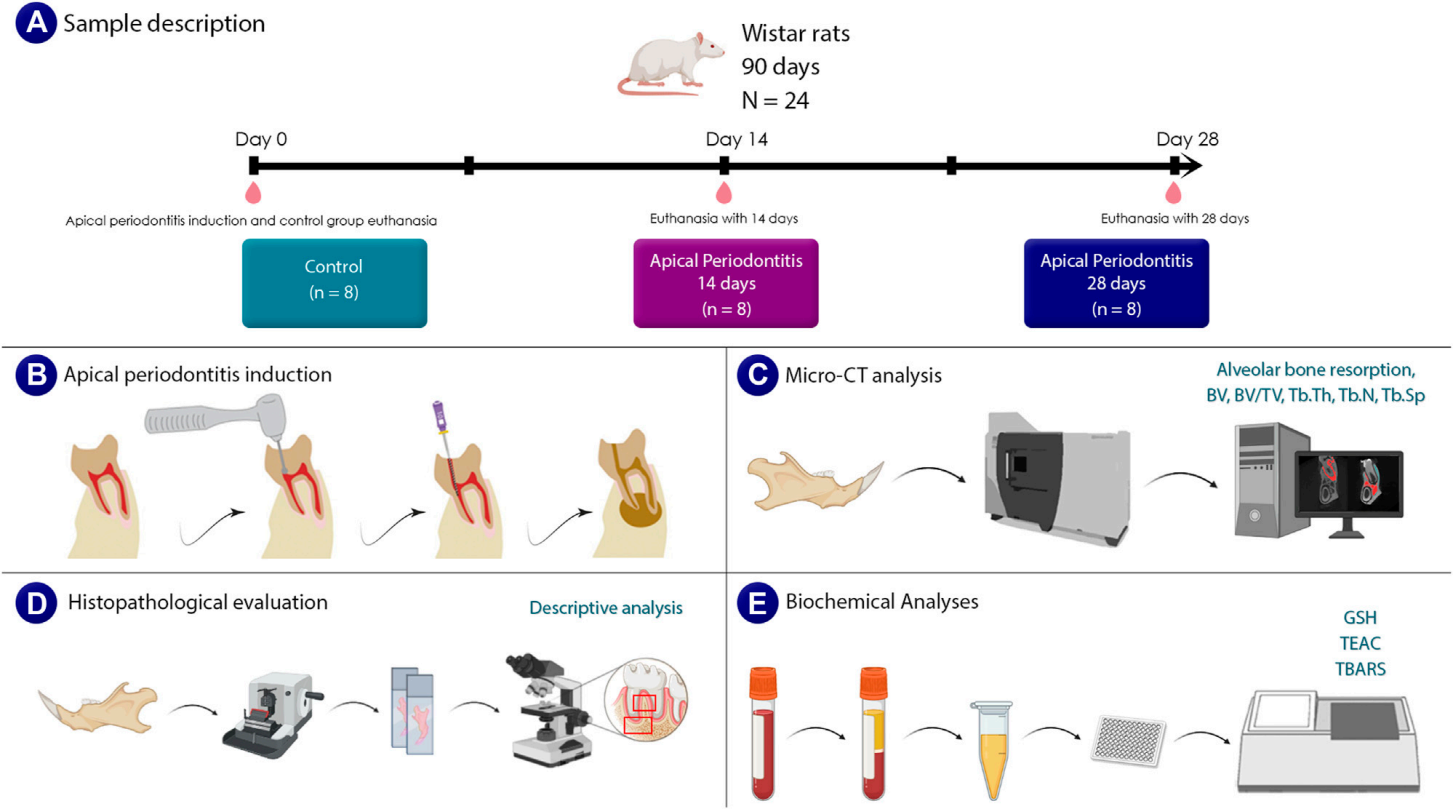
Sample description and experimental steps. **(A)** shows a sample description that includes information on the age, gender, and number of animals. **(B)** shows the steps involved in inducing apical periodontitis. A schematic illustration of the carried out micro-tomographic analyses is shown in **(C)**. The steps needed for a histological evaluation are shown in **(D)**. Finally, in **(E)**, an illustrative representation of the analysis of oxidative biochemistry and the investigated parameters are presented.

Throughout the study, we monitored the body weight of the animals every week. After 14 and 28 days, the animals were sedated using ketamine hydrochloride (90 mg/kg) and xylazine hydrochloride (9 mg/kg), until we ensure the absence of corneal reflexes. Blood samples were collected via a 5-mL syringe from the heart of the animals for oxidative biochemistry assessment. Subsequently, the animals underwent perfusion fixation by administering 0.9% heparinized saline solution followed by 4% formaldehyde through the left ventricle. The hemimandibles were carefully dissected from both sides using a scalpel and surgical scissors for micro-computed tomography (micro-CT) and histopathological analysis. For a visual representation of the sample description and experimental steps, refer to Figure 1.

### 2.3 Micro-computed tomography (micro–CT) analysis

For the micro-CT evaluation, the left hemimandible was fixed in 4% formaldehyde in a liquid volume at least 30 times larger than the piece. Then, the hemimandibles were submitted to micro-CT (MicroCT.SMX-90 CT; Shimadzu Corp., Kyoto, Japan). Images were captured under a rotation of 360°, with an intensity of 70 kV and 100 mA. After this, images were reconstituted by inspeXio SMX-90CT software (Shimadzu Corp., Kyoto, Japan), with a voxel size of 10 µm and resolution of 1024 × 1024, which resulted in 541 images per sample. All the datasets were exported using the Digital Imaging and Communications in Medicine (DICOM) file.

The area of alveolar bone resorption was reconstructed using CTAn software (V1.15.4.0; Bruker, Kontich, Belgium). The hemimandibles were placed in a standard position (Figure 1), where the periodontal ligament could be observed through the coronal section. Then, the reconstructed volume of interest (VOI) included the periodontal ligament space and the destruction around the root, just as described by Chen et al. (2019) The examiner then manually delimited the destruction zone volume in all the mandibular first molar roots, beginning with the mesial root and ending with the distal root. Each VOI root started with the first coronal slice of the mesial root, which was surrounded by the bone crest, and continued to the distal region, ending when it reached the mandibular second molar.

The CTAn software (V1.15.4.0; Bruker, Kontich, Belgium) was also used on a set of 220 images from the inferior first molar bone region to verify the alveolar bone quality. Then, the alveolar bone surrounding the first molar roots was included in the region of interest (ROI). The examiner manually delimited the bone in each coronary plane from the point closest to the mesial root to the point farthest from the distal root. To differentiate cortical bone, trabecular bone, and bone marrow, the CTAn software adjusted the applicable threshold following the guidelines provided by the manufacturer, with a threshold (31–71) applied to the segmentation of the different scores of gray color present in the image. Finally, on the reminiscent bone that was not affected by the lesion, the following parameters were measured: reminiscent bone volume (BV), percent of bone volume (BV/TV), trabecular thickness (Tb.Th), trabecular number (Tb.N), and trabecular spacing (Tb.Sp).

### 2.4 Histopathological evaluation

The other hemimandibles of each animal were collected and post-fixed for 24 h in 4% formaldehyde. Then, for 90 days, the samples were demineralized in 10% ethylenediaminetetraacetic acid (EDTA). The pieces were then dehydrated in alcohol, diaphanized in xylene, and embedded in paraplast. Following inclusion, the materials were sliced on a Leica RM 2045 microtome (Leica Microsystems, Nussloch - Germany) with a thickness of 5 µm in the mesiodistal orientation and placed on individual slides. Sections were stained with hematoxylin and eosin and photomicrographed with a digital color camera (Cyber-Shot DSC W-230, 4X optical zoom, Sony, Tokyo, Japan) attached to an optical microscope (Leica QWin Plus-Leica Microsystems, Nussloch - Germany).

The inflammatory profile of periapical lesions was diagnosed in semi-serial sections along the entire length of the mandible. The severity of the lesion was then determined based on the intensity, characteristics, and extension of the inflammatory infiltration, the preservation of the cementum, and the integrity of the bone.

### 2.5 Biochemical analyses

#### 2.5.1 Sample preparation

Before prefunding the animals, blood samples were collected in tubes containing 50 μL of 5% ethylenediaminetetraacetic acid (EDTA) and centrifuged for 10 min at 3,000 rpm. Following centrifugation, plasma was collected and stored in Eppendorf tubes at −80°C for further analysis of reduced glutathione (GSH) levels, Trolox equivalent antioxidant capacity (TEAC), and levels of thiobarbituric acid reactive substances (TBARS) (Miranda et al., 2018).

#### 2.5.2 Reduced glutathione content (GSH)

The GSH level measurements were determined using a modified Ellman method (Ellman, 1959). This approach is based on the inherent ability of glutathione to catalyze the conversion of 5,5-dithiobis-2-nitrobenzoic acid (DTNB) to nitrobenzoic acid (TNB). The difference in absorbances (T3-T0) is proportional to the concentration of GSH, where T0 is the absorbance measurement immediately after adding DTNB to the sample and T3 is the value taken 3 min later. The GSH levels are expressed as μmol/mL.

#### 2.5.3 Trolox equivalent antioxidant capacity (TEAC)

The method used to assess TEAC was described by Rufino et al. (2007) This is a method for determining the total antioxidant activity of body fluids that is not specific. In this experiment, the solution with ABTS + radical was added to the phosphate buffer (PBS; pH 7.2) until obtaining an absorbance of 0.7 ± 0.02 at 734 nm. Then, 30 μL of plasma or Trolox standards (standard curve) were added to 2,970 μL of ABTS solution, and the absorbance was measured 5 min later. Absorbances were tested in triplicate and calculated using a standard curve that included Trolox as a standard (Re et al., 1999) The total antioxidant capacity of plasma is expressed in mmol/L.

#### 2.5.4 Thiobarbituric acid reactive substances (TBARS)

The analysis of thiobarbituric acid reactive substances (TBARS) was performed as an indicator of lipid peroxidation as previously described (Kohn and Liversedge, 1944; Percário, 1994). The malondialdehyde (MDA) formed during the lipid peroxidation process interacts with thiobarbituric acid (TBA) to form chromophore material, measured in the organic phase of the reaction by spectrophotometry at 535 nm, the concentration of MDA is expressed in nmol/mL.

### 2.6 Statistical analyses

The Shapiro-Wilk test analyzed the normality of the data distribution. Then, statistical analyses were performed by one-way ANOVA followed by the Tukey test. Moreover, for bone quality and oxidative biochemistry evaluation, the multivariate analysis of variance (MANOVA) was executed to embrace all parameters. Bodyweight data was evaluated by repeated measure two-way ANOVA. All results were expressed as mean ± standard error of the mean (SEM). Statistical differences were adopted when *p* < 0.05. GraphPad Prism 9.0. software (GraphPad, San Diego, CA, United States of America) and Jamovi version 2.3 (www.jamovi.org) (Jamovi, 2019; R Core Team. R, 2013; Jarek, 2009) was employed for statistical analyses. A descriptive analysis was performed for histopathologic evaluation.

## 3 Results

### 3.1 Bodyweight evaluation

**FIGURE 2:**
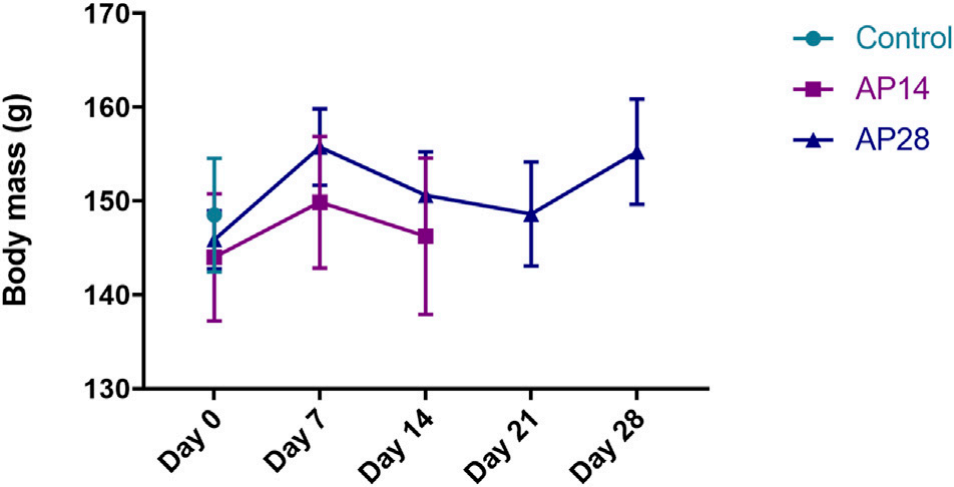
Body mass evaluation among the groups. Results are expressed as mean ± standard error for control, apical periodontitis at 14 days (AP14), and apical periodontitis at 28 days (AP28). Two-way ANOVA.

Mean body weight did not show statistically significant differences among groups and during the periods evaluated in this study (*p* = 0.42), as shown in Figure 2.

### 3.2 Micro-computed tomography (micro–CT) analysis

#### 3.2.1 Alveolar bone resorption

**FIGURE 3:**
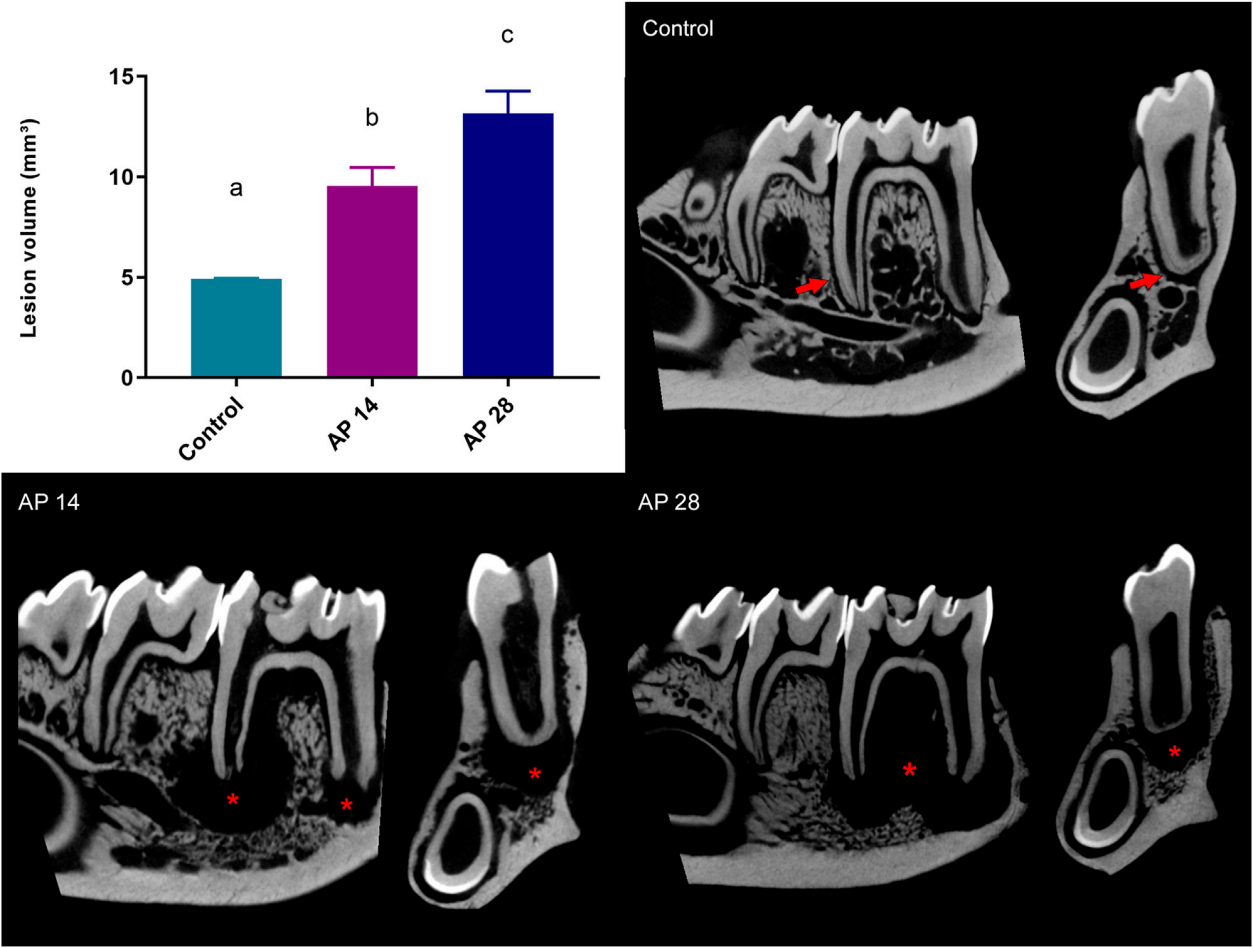
Alveolar bone resorption in all experimental groups. Results are expressed as mean ± standard error of the lesion volume, representing the alveolar bone resorption mm³. AP28 group presented the highest alveolar bone resorption identified by lesion volume. The same letters indicate no significant differences between groups (*p* > 0.05). ANOVA followed by Tukey *post hoc* test (n = 8). The right panel shows the reconstruction of the hemimandibles of the control group. The arrows indicate preserved bone integrity; AP14 shows apical periodontitis in the 14 days group, and AP28 shows apical periodontitis in the 28 days group. The asterisks (*) represent the regions of bone resorption.

Hemimandible analyses show that animals of the apical periodontitis at 14 days group had a higher bone destruction volume than the control group (9.54 ± 0.03 vs. 4.93 ± 0.93; *p* = 0.0089). Moreover, AP in 28 days had an increase in alveolar bone resorption compared to apical periodontitis in 14 days (13.17 ± 1.10 vs. 9.54 ± 0.03; *p* = 0.03) and control group (13.17 ± 1.10 vs. 4.93 ± 0.93; *p* = 0.0002), as shown in Figure 3.

#### 3.2.2 Alveolar bone quality

Compared to the control, the animals at 14 and 28 days had lower bone volumes (14.06 ± 0.93 vs. 10.10 ± 1.17 vs. 9.95 ± 0.59; *p* < 0.05; respectively). However, apical periodontitis at 14 days (AP14) and at 28 days (AP28) had no significant statistical difference between each other (10.10 ± 1.17 vs. 9.95 ± 0.59; *p* = 0.99) (Figure 5B).

Regarding the percent of bone volume (BV/TV), compared to control animals, the AP28 group showed a significant reduction (79.3% ± 4.55% vs. 65.10% ± 3.12; *p* = 0.03). Interestingly, apical periodontitis at 14 days (AP14) did not differ statistically when compared to the control (70.7 ± 2.2 vs. 79.3 ± 4.55; *p* = 0.24) and the AP28 group (70.7 ± 2.2 vs. 65.10 ± 3.12; *p* = 0.49) (Figure 5C).

The control group presented the highest trabecular thickness parameter (Tb.Th) values (0.33 ± 0.02). Furthermore, even after 14 and 28 days, apical periodontitis exposure resulted in a decrease in Tb.Th (*p* < 0.05). However, apical periodontitis at 28 days showed intensified loss of thickness compared to the 14 days group (0.19 ± 0.01 vs. 0.25 ± 0.009; *p* = 0.03) (Figure 5D).

In addition, in contrast to Tb.Th data, the number of trabeculae (Tb.N) was higher in apical periodontitis groups compared to the control (2.34 ± 0.19; *p* < 0.05). Despite this, there was no statistically significant difference in trabecular number between apical periodontitis at 14 days and 28 days (3.03 ± 0.05 vs. 2.95 ± 0.16; *p* = 0.91) (Figure 5E).

**FIGURE 4:**
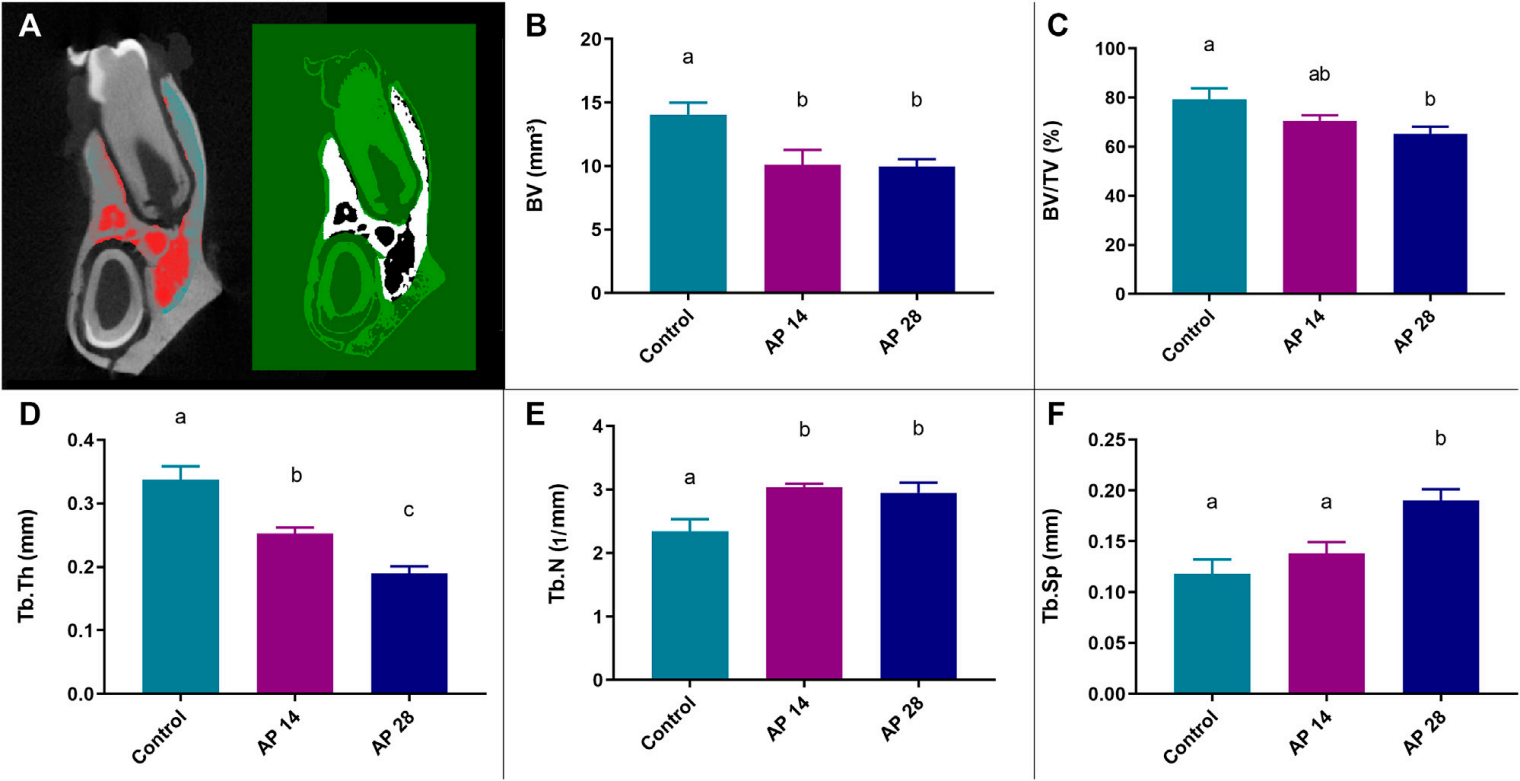
Results from the micro-CT quality analysis obtained through the CTAn software (V1.15.4.0; Bruker, Kontich, Belgium). **(A)** Region of interest (ROI) used for evaluation. **(B)** Bone volume (BV) parameter in all experimental groups. **(C)** Percent of bone volume compared to total tissue volume (BV/TV). **(D)** Trabecular thickness parameter (Tb.Th). **(E)** Trabecular number parameter (Tb.N). **(F)** Trabecular spacing parameter (Tb.Sp). For these results, the same letters indicate no significant differences between groups (*p* > 0.05). ANOVA followed by Tukey *post hoc* test was used (n = 8).

**FIGURE 5:**
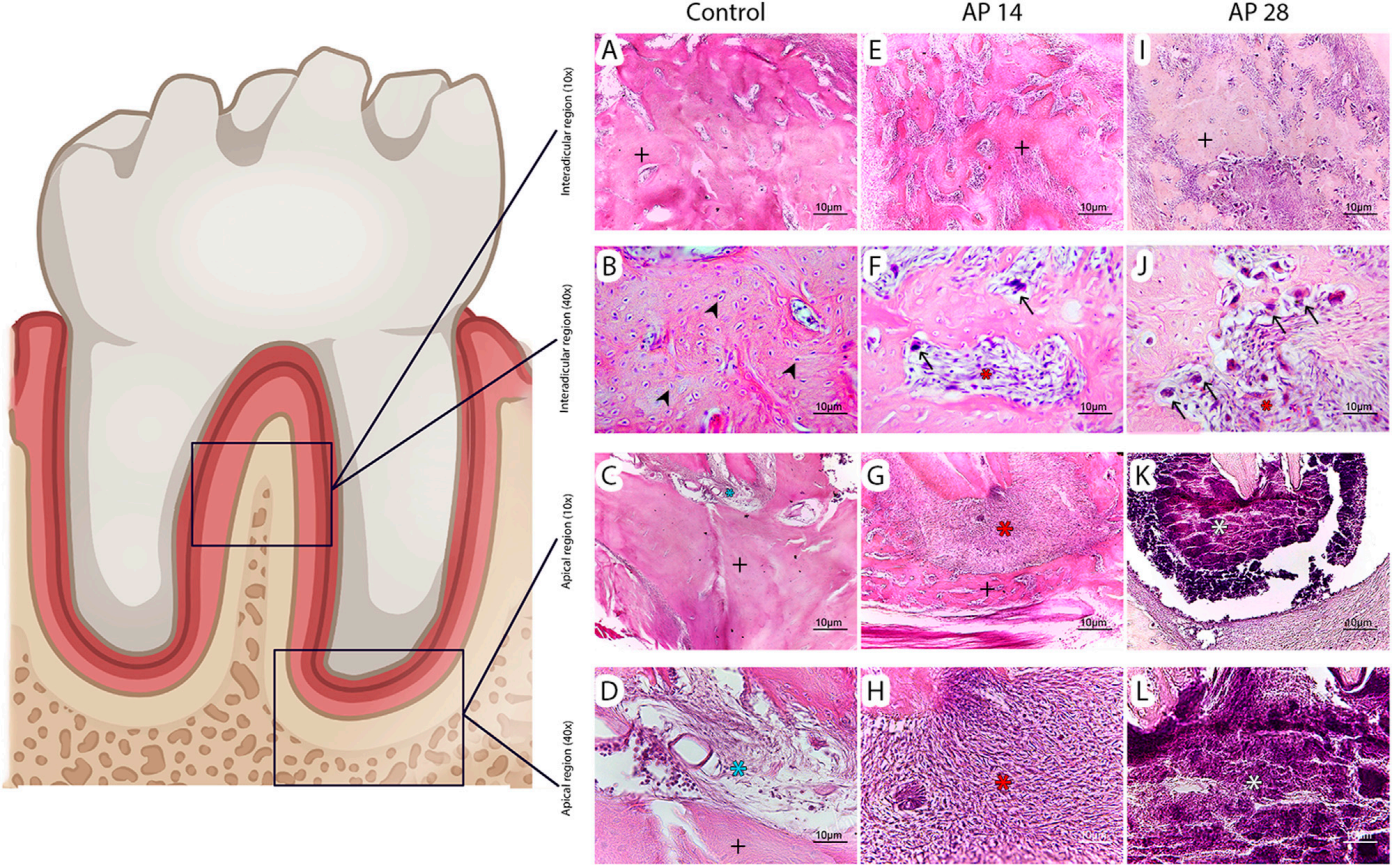
Histopathological analysis. According to the descriptive analysis, the control group exhibited no inflammation and intact alveolar bone tissue **(A–D)**. The 14-day apical periodontitis group (AP14) showed isolated patches of resorbed alveolar bone in the furcation area **(E and F)** and a considerable inflammatory infiltration in the periapical region **(G and H)**. The group with apical periodontitis at 28 days (AP28) exhibited more severe inflammatory infiltration **(I–L)** and the presence of necrotic tissue in the periapex (K and L). Photomicrographs **(A, E, and I)** depict the interradicular region of the three groups at ×10 magnification, while **(B, F, and J)** represent the same region at ×40 magnification. **(C, G, and K)** show the periapical area of the groups at ×10 magnification, and **(D, H, and L)** display the periapical region at a higher magnification of 40x, closer to the root apex. The symbols used are as follows: Alveolar bone (black**+**), osteocytes (arrowhead), osteoclasts (arrow), inflammatory tissue (red*), necrotic tissue (white*), and connective tissue (blue*).

Finally, the trabecular spacing (Tb.Sp) was intensely increased in apical periodontitis at 28 days compared to both control (0.19 ± 0.11 vs. 0.11 ± 0.01; *p* = 0.004) and apical periodontitis at 14 days group (0.19 ± 0.11 vs. 0.13 ± 0.01; *p* = 0.03). Interestingly, AP14 demonstrated no significant difference when compared to the control (0.13 ± 0.01 vs. 0.11 ± 0.01; *p* = 0.51) (Figure 5F). The graphical representation of all quality parameters is shown in Figure 4.

In addition, the multivariate analysis of variance (MANOVA) provided a regression analysis and analysis of variance for the alveolar bone variables. The result of Pillai’s Trace test is shown in Table 1.

**TABLE 1:** MANOVA for alveolar bone quality paraments and oxidative biochemistry using Pillai’s Trace test.

Alveolar bone quality						
Multivariate Tests						
		value	F	df1	df2	p
Groups	Pillai’s Trace	1.37	5.40	8	20	0.001
Oxidative biochemistry						
Multivariate Tests						
		value	F	df1	df2	p
Groups	Pillai’s Trace	1.17	7.48	6	32	<.001

df1: degrees of freedom associated with the groups being compared for both alveolar bone quality and oxidative biochemistry parameters.

df2: degrees of freedom associated with the error term.

### 3.3 Histopathological evaluation

In the control group (Figures 5A–C), no inflammation was observed in the periapical region. In contrast, the 14-day apical periodontitis group (Figures 5D–F) exhibited moderate mononuclear infiltration throughout the alveolar bone in the periapical region, with this infiltrate extending to the interradicular area.

Remarkably, in the 28-day apical periodontitis group (Figures 5G–I), a significantly more intense and extensive inflammatory infiltrate was observed. Furthermore, localized areas of severe inflammatory infiltration were present throughout the alveolar bone of the interradicular region. These areas displayed necrosis and pronounced inflammation, highlighting the progression of the lesion compared to the 14-day group.

### 3.4 Biochemical analyses

**FIGURE 6:**
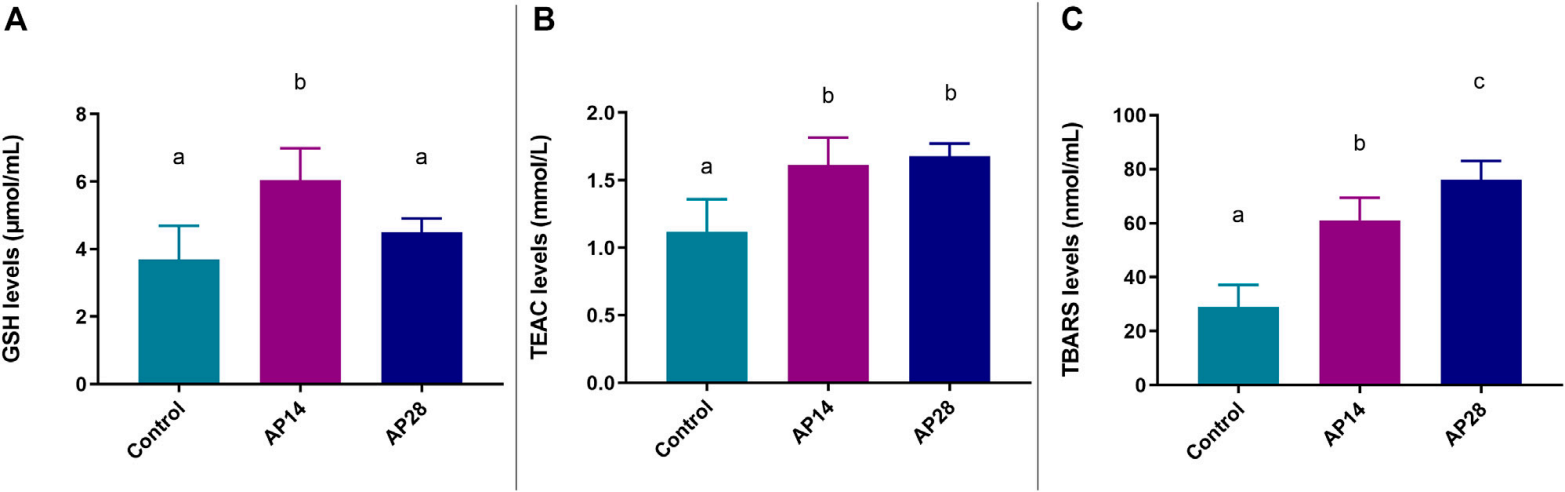
Biochemical evaluation. Results are expressed as mean ± standard error of the levels of each biochemical parameter: GSH **(A)**, TEAC **(B)**, and TBARS **(C)**. In panel A, GSH is lower on day 28 than on day 14, while in panel C, TBARS is higher on day 28 than on day 14, suggesting an oxidative stress. The same letters indicate no significant differences between groups (*p* > 0.05). ANOVA followed by Tukey *post hoc* test (n = 8).

Firstly, in relation to reduced glutathione (GSH) levels, the AP14 group showed increased GSH levels compared to the control (6.05 ± 0.35 vs. 3.69 ± 0.57; *p* = 0.0016) and the AP28 group (6.05 ± 0.35 vs. 4.50 ± 0.15; *p* = 0.0058). However, the control and AP28 groups were not statistically significant (3.69 ± 0.57 vs. 4.50 ± 0.15; *p* = 0.31). All biochemical results are shown in Figure 6.

Additionally, regarding the antioxidant capacity levels (TEAC), apical periodontitis at 14 and 28 days did not show statistical significance between them (1.61 ± 0.07 vs. 1.68 ± 0.03; *p* = 0.73). However, their results were significantly higher compared to the control group (1.12 ± 0.12 vs. *p* < 0.05).

In addition, the AP28 group had higher levels of TBARS compared to both AP14 (76.1 ± 2.44 vs. 61.1 ± 2.97; *p* = 0.0033) and control group (76.1 ± 2 .44 vs. 29.0 ± 4.05; *p* < 0.0001), as shown in Figure 6C. Furthermore, the AP14 group had higher TBARS levels than the control (61.1 ± 2.97 vs. 29.0 ± 4.05; *p* < 0.0001).

After that, the multivariate analysis of variance (MANOVA) revealed that there is a significant difference when evaluating all oxidative parameters as dependent variables (Table 1).

## 4 Discussion

Our findings were the first to demonstrate that blood oxidative biochemistry is modulated by the duration and severity of the periapical injury since the oxidative biochemistry reaction varied over time. This occurred by reducing the antioxidant response mediated by GSH and increasing lipid peroxidation proportionally to bone destruction. Secondly, we demonstrated that, after 14 days of apical periodontitis, rats exhibited significant inflammatory reactions and bone destruction, prompting a systemic biochemical response.

The literature points out different protocols of induction of apical periodontitis in experimental models, such as access and inoculation of bacteria with and without sealing (Yamauchi et al., 2011; Palma et al., 2017) and pulp exposure with or without cavity sealing (Sehirli et al., 2019; Tibúrcio-Machado et al., 2021; Aksoy et al., 2019; Verma et al., 2017; da Silva et al., 2010). These experimental models were performed on different animals such as dogs (Yamauchi et al., 2011; da Silva et al., 2010; Wang et al., 2010), monkeys (MÖLLER et al., 1981; FABRICIOUS et al., 1982; FABRICIUS et al., 1982), ferrets (Verma et al., 2017) and rats (Wang and Stashenko, 1991; Brilhante Wolle et al., 2012; Prieto et al., 2017; Aksoy et al., 2019; Dal-Fabbro et al., 2019; Sehirli et al., 2019; Cosme-Silva et al., 2020; Chen et al., 2021; Tibúrcio-Machado et al., 2021). In this work, we chose to induce apical periodontitis in rats by accessing the pulp and leaving it open to the oral environment. Previous studies have widely described this well-established experimental model (Aksoy et al., 2019; Dal-Fabbro et al., 2019; Sehirli et al., 2019; Cosme-Silva et al., 2020; Tibúrcio-Machado et al., 2021).

The choice of different time points in this study was based on literature reports indicating significant increases in periapical lesion volume between 14 and 21 days, followed by stability until 28 days of pulp exposure (Minhoto et al., 2021). The progression of the lesion can be divided into an active or developing phase from day 0 to day 15, and a chronic phase from days 20–30 (Wang and Stashenko, 1991). The progression of the periapical lesion at two distinct periods results in different reactions. Micro-CT scans showed that the lesions were visible after 14 days and reached their maximum size after 28 days (Wang and Stashenko, 1991), demonstrating a correlation between resorbing activity and lesion expansion (Wang and Stashenko, 1991; Tani‐Ishii et al., 1995). Thus, our study found that by 14 days, there was already a well-established apical lesion with an inflammatory profile and bone damage capable of generating a systemic oxidative response. Micro-CT evaluations revealed that the lesion volume was smaller at 14 days compared to 28 days, with changes in trabecular thickness and number. Histological examination indicated ongoing bone resorption, likely due to the presence of osteoclasts and mononuclear infiltrates. These findings suggest a relationship between the number of osteoclasts and the higher posterior lesion volume (Sun et al., 2021).

Hence, after 28 days, a higher degree of periapical bone resorption and changes in certain indicators of bone quality was observed, consistent with previous studies (Balto et al., 2000; Chen et al., 2019). The remaining bone volume and the bone volume to tissue volume ratio were similar to those at 14 days, although the BV/TV showed a significant decrease compared to the control. Additionally, there was a notable decrease in trabecular thickness after 28 days, potentially leading to increased trabecular spacing. The histopathological examination further confirmed these findings, demonstrating areas of more pronounced inflammatory infiltrate and necrosis, as well as significant loss of interradicular and apical alveolar bone.

Many research studies have focused on investigating biomarkers associated with periapical injury and their relationship with systemic diseases (Inchingolo et al., 2013; Kimak et al., 2015; Gomes et al., 2018; Sirin et al., 2021). Interleukins and tumor necrosis factors have been found to play a role in the pathogenesis of chronic inflammatory diseases like pulpitis and apical periodontitis (Tani‐Ishii et al., 1995). Various biomarkers, such as IL-10, TNF2, TNFB, and bacterial components (LPS), have been identified in infected root canals and periapical lesions, triggering periapical bone resorption (Wang and Stashenko, 1991; Kawashima and Stashenko, 1999). Reactive oxygen species (ROS) have also been linked to bone health, inhibiting osteoblast differentiation and increasing osteoclastogenesis, leading to bone resorption (Wauquier et al., 2009; Hernández-Ríos et al., 2017; Sies et al., 2017; Vengerfeldt et al., 2017). RANKL, expressed by osteoblasts, plays a critical role in osteoclast differentiation and subsequent bone resorption. Oxidative stress disrupts this bone interaction, decreasing osteoblast-mediated bone formation and increasing osteoclast activities and bone resorption (Wauquier et al., 2009). As observed in our study, at 28 days, an increase in bone loss, necrosis, and inflammation, along with a decline in GSH and an increase in lipid peroxidation, indicated a redox imbalance.

In this study, we investigated three important parameters that have considerable applicability in clinical research: GSH, TEAC, and TBARS (Spanidis et al., 2016; Dumitrescu et al., 2017). Glutathione is the most abundant antioxidant in this system and serves as the primary regulator, controlling the inflammatory process (Bains and Bains, 2015). As a result of the periodontal damage associated with the loss of this antioxidant, the inflammatory reaction becomes more severe. We found a rise in reduced glutathione levels in the AP14 group in response to apical periodontitis, which might indicate a significant procedure to the inflammatory process and/or response. However, the AP28 group did not maintain this increase since it did not exhibit a statistically significant difference from the healthy controls. These findings suggest a limited response to the long-term maintenance of high glutathione levels.

The potential of antioxidant capacity as a predictor of oral cavity diseases has been investigated (Öngöz Dede et al., 2016). Plasma antioxidant capacity, measured using the TEAC assay, showed elevated levels after inducing apical periodontitis at 14 and 28 days. Although the measurement of plasma antioxidant capacity lacks specificity, a reduction in plasma antioxidant capacity suggests oxidative stress, while an increase may indicate an early response to inflammation (Brock et al., 2004). The reduction of enzymatic antioxidants such as glutathione reductase occurs concomitantly with the progression of chronic inflammation, thereby restricting the capacity to mend the damage caused by free radicals. Our study revealed a substantial and time-dependent elevation in lipid peroxidation, suggesting a decline in the capacity of the body to counteract oxidative stress and an amplified inflammatory reaction that produces reactive oxygen species. However, TEAC levels were higher and comparable to the AP14 group, implying that antioxidant defense can be modulated through alternative pathways apart from glutathione. Moreover, with the progression of the inflammatory process towards a chronic state, there could be a decrease in the levels of enzymatic antioxidants, resulting in an elevation of lipid peroxidation levels and a decline in the capacity to mend the damage caused by free radicals.

Nevertheless, this investigation presents some limitations. First, because this is an animal study, the results cannot be extrapolated to humans. Thus, there is a need for clinical studies verifying oxidative biochemistry before and after endodontic treatment, and also comparing it to healthy patients. In addition, we checked the systemic biochemistry using only the blood of the rats. Finally, we explored only three of the various parameters to investigate the oxidative response.

Therefore, our findings stimulate new investigations regarding the response of other antioxidant parameters, such as the antioxidant enzymes superoxide dismutase (SOD), responsible for converting the superoxide anion (O_2_^−^) into hydrogen peroxide (H_2_O_2_), and catalase (CAT), responsible for removing H_2_O_2_. Also, it is essential to investigate the modulation of other oxidative stress parameters, such as damage to nucleic acids, proteins, and carbohydrates. Besides, clinically our findings encourage further research into the remission of this condition by endodontic treatment and the duration of this remission. At the same time, our study suggests that using antioxidants as an adjuvant therapy strategy, as is done in chronic periodontitis (Castro et al., 2019), may reduce the biochemical changes caused or possibly interfere with the progression of apical periodontitis.

## 5 Conclusion

Our findings indicate that oxidative biochemistry parameters are associated with different stages of periapical lesions. After 14 days, there was a decrease in reduced glutathione levels, which was associated with a mononuclear inflammatory response and an increase in lipid peroxidation, indicating that cellular damage had already occurred. As a result of this mechanism, an osteoclast activation response occurred later in the process, resulting in more significant periapical bone loss, which may be related to a decrease in reduced glutathione and an increase in lipid peroxidation.

## Data Availability

The original contributions presented in the study are included in the article/supplementary material, further inquiries can be directed to the corresponding author.
